# Assessing Callous-Unemotional Traits in Chinese Detained Boys: Factor Structure and Construct Validity of the Inventory of Callous-Unemotional Traits

**DOI:** 10.3389/fpsyg.2019.01841

**Published:** 2019-08-07

**Authors:** Xintong Zhang, Yiyun Shou, Meng-Cheng Wang, Chuxian Zhong, Jie Luo, Yu Gao, Wendeng Yang

**Affiliations:** ^1^Department of Psychology, Guangzhou University, Guangzhou, China; ^2^The Center for Psychometrics and Latent Variable Modeling, Guangzhou University, Guangzhou, China; ^3^Research School of Psychology, The Australian National University, Canberra, ACT, Australia; ^4^The Key Laboratory for Juveniles Mental Health and Educational Neuroscience in Guangdong Province, Guangzhou University, Guangzhou, China; ^5^School of Psychology, Guizhou Normal University, Guiyang, China; ^6^Brooklyn College, The City University of New York, New York, NY, United States

**Keywords:** callous-unemotional traits, psychopathy, detained juvenile, factor structure, confirmatory factor analysis, validation

## Abstract

The Inventory of Callous-Unemotional Traits (ICU) was designed to evaluate multiple facets of Callous-Unemotional (CU) traits in youths. However, no study has examined the factor structure and psychometrical properties of the ICU in Chinese detained juveniles. The current study assesses the factor structure, internal consistency and convergent validity of the ICU in 613 Chinese detained boys. Confirmatory factor analysis results indicated that the original three-factor model with 24 items showed an unacceptable fit to the data, however, the 11-item shortened version of the ICU (ICU-11) with callousness and uncaring dimensions showed the best fit. Moreover, the ICU-11 total score and factor scores had good and acceptable internal consistencies. The convergent and criterion validity of the ICU-11 was demonstrated by comparable and significant associations in the expected direction with relevant external criteria (e.g., psychopathy, aggression, and empathy). In conclusion, present findings indicated that the ICU-11 is a reliable and efficient instrument to replace the original ICU when assessing CU traits in the Chinese male detained juvenile sample.

## Introduction

The Callous-Unemotional (CU) traits in children and adolescents are a specifier of the criteria for conduct disorder (CD) in the fifth edition of the Diagnostic and Statistical Manual of Mental Disorders (DSM-5, American Psychiatric Association, 2013), and are considered as an affective characteristic of psychopathic personality disorder (Frick and Moffitt, 2010). And the CU traits have been proven to be the most crucial predictors of criminal activities (Asscher et al., 2011). Features of a high level of the CU traits include a lack of concern about performance, shallow emotions, a lack of empathy and guilt, and having low sensitivity to others’ feelings (Frick, 2009). As such, the CU traits may be used to define a subgroup of youths with severe and persistent conduct problems, delinquency, or aggression particularly referring to a more proactive type of aggression (Kahn et al., 2012; Byrd et al., 2013). Different from other antisocial juveniles, those with CU traits tend to have difficulty in dealing with negative emotional stimuli (Kimonis et al., 2008), a lack of fearful inhibitions and anxiety (Frick et al., 1999) and a lack of sensitivity to punishment cues (Fisher and Blair, 1998). Remarkably, psychopathy is one of the most important predictors of criminality (DeLisi and Vaughn, 2015; DeLisi, 2016; DeLisi et al., 2018). Substantial evidence has demonstrated that the juvenile with higher psychopathy especially those have affective deficits and less self-control, had increased likelihood of engaging in violent forms of antisocial behaviors (DeLisi et al., 2010, 2018), in criminal careers that continue into the adulthood (Vaughn and DeLisi, 2008).

Understanding CU traits in delinquent and antisocial adolescents requires efficient, reliable and valid measurement tools. The Inventory of Callous-Unemotional Traits (ICU) was developed as a stand-alone and comprehensive self-report instrument (Frick, 2004). The ICU contains 24 items that are expanded from the CU factor (four items) of the Antisocial Process Screening Device (APSD; Frick and Hare, 2001). Since its introduction, various informant versions of the ICU have been increasingly endorsed in research, and have demonstrated reliable associations with external criteria variables in both incarcerated and community youth (Roose et al., 2010; Pihet et al., 2015; Pechorro et al., 2016b, 2017). However, a recent meta-analysis by Deng et al. (2019) has noted that there remains a lack of evidence of the applicability of the ICU among non-European-American samples. Although there has been an attempt of validating the ICU among Chinese community samples (Wang et al., 2017b, 2019), little is known of the utility of the ICU in clinical settings in non-English-speaking delinquent populations.

Furthermore, although the ICU was originally developed as a unidimensional measure of CU traits (an overarching CU factor containing three subfactors: unemotional, callousness and uncaring), this early proposed three-factor, as well as a three-factor bifactor model (Essau et al., 2006), received limited support in either community (Ciucci et al., 2014; Wang et al., 2017b, 2019) or delinquent samples (e.g., Kimonis et al., 2008) due to the poor overall fit of these models. Notably, the unemotional factor has been shown to have relatively poor psychometric properties, showing low reliability, poor factor loadings and inadequate correlations with external criteria (e.g., Essau et al., 2006; Kimonis et al., 2008; Byrd et al., 2013). Many recent studies have excluded some or all of the unemotional factor items, and have focused on developing a range of short versions of the ICU.

For example, Hawes et al. (2014) developed a 12-item shortened form of the ICU (ICU-12) using item response theory. The ICU-12 has two correlated factors: callousness (seven items) and uncaring (five items), and its validity and reliability were supported in a number of subsequent studies that used detained samples (e.g., Colins et al., 2016; Paiva-Salisbury et al., 2017). Two recent studies found that an 11-item model (ICU-11) which excluded the item, “I do not show my emotions to others” – the only item retained from the unemotional factor – achieved a better fit than the ICU-12 among Chinese-speaking samples using university students (Wang et al., 2017b) and community children (Wang et al., 2019). This is possibly due to the fact that expressing emotion is generally not encouraged in Chinese culture, thus resulting in the low discriminability of the item among Chinese populations. Nevertheless, the ICU-11 displayed measurement invariance across informants and occasions and had strong evidence for its criteria validity (Wang et al., 2019). The results of Wang et al. (2017b) also showed strong associations with other measures of psychopathic traits, and both of the two factors (callousness and uncaring) correlated significantly with the total scores on the ASPD and proactive aggression.

Psychopathy has been integrated into mainstream criminological theories (DeLisi and Vaughn, 2015), and at least in part, explains the causal mechanisms underlying chronic, serious, and violent delinquent trajectories, so that psychopathy can be used as a risk for the development and maintenance of delinquent behaviors (Asscher et al., 2011; Corrado et al., 2015). Moreover, regardless the intensity of the violence, the CU traits were found significantly correlated with violent offending (Sherretts et al., 2017). Despite the evidence for the validity and reliability of the short versions of the ICU among Chinese community samples, the results may not be generalized to clinical and detained populations. Given that the gravity of juvenile crimes has aggravated in recent years in mainland China, which society has paid more and more attention to, and CU traits are a clinical construct, it is important to expand upon previous findings among different Chinese samples, particularly in detained youths, and test other relevant correlates such as empathy and additional instruments of psychopathic features.

### The Current Study

The main purpose of this study was to explore the factor structure of the ICU in a sample of Chinese detained juveniles. Confirmatory factor analyses (CFA) were conducted to compare various factor structures proposed in previous studies. Based on findings from recent studies (Wang et al., 2017b, 2019), we hypothesized that the ICU-11 with the callousness and uncaring dimensions would be the best fit for the data.

The second purpose of this study was to evaluate the psychometric properties of the best-fitted model (ICU-11) including internal consistency and convergent validity. Based on previous research (Wang et al., 2017b, 2019; Deng et al., 2019), it was expected that the ICU-11 would have satisfactory internal consistency while keeping sufficient information from the original 24-item version of the ICU. Additionally, we expected that the ICU-11 scores would correlate positively with alternative instruments of the psychopathic traits (i.e., the Antisocial Process Screening Device – Self-Report Version [APSD-SR] and the Youth Psychopathic Traits Inventory – Short Version [YPI-S]), and the instrument that measures reactive and proactive aggression. Conversely, we expected the scores of the ICU-11 to correlate negatively with empathy (Kimonis et al., 2013). Based on previous findings using indicators of the offending history (Byrd et al., 2013; Pechorro et al., 2017), we expected that the ICU-11 would have correlations with several external criterion variables including the participants’ age, age of incarceration into a juvenile detention center and the duration of incarceration (i.e., difference between current age and first arrest age).

## Materials and Methods

### Participants

The current study included juvenile male participants recruited from the Guangdong Juvenile Detention Center. Excluding participants who had intellectual disability, a total of 613 male participants (*N* = 613, mean age = 17.14, SD = 1.09, range = 14–22) participated voluntarily in the study. Participants were predominantly from nuclear families (*N* = 466, 76.0%), followed by single-parent families (*N* = 135, 22.0%); 79.1% (*N* = 485) came from a multiple-child family. About 64.6% participants (*N* = 396) reported that they had lived with their parents before the age of twelve, followed by grandparents (*N* = 158, 25.8%) and finally, relatives (*N* = 24, 3.9%). With regard to their parents’ level of education, 88% of participants’ fathers and 92.3% of their mothers were at or below senior secondary school level (similar to Grade 12 in United States). The mean age of participants’ first incident of arrest was 15.49 years (SD = 0.87 years). Within the sample, the most common offence committed was robbery (*N* = 411, 67.0%), followed by physical assault (*N* = 70, 11.4%) and sexual assault (*N* = 50, 8.2%)

### Procedure

After receiving written informed consent from the detainees’ parents or caregivers, the detainees were informed about the aims, content and duration of the study by trained research assistants. They were informed that participation was voluntary, and completion of the study was anonymous. The participants completed the paper-and-pencil self-report survey during their classes, each of which contained 35–40 inmates under the supervision of the research assistants. During the study, participants were allowed to ask for clarification if they did not understand any part of the questionnaire. The study duration was approximately 40 min. This study was approved by the Human Subjects Review Committee at the Guangzhou University. Written informed consent was obtained from all adult participants and from the parents/legal guardians of all non-adult participants.

### Measures

#### Inventory of Callous-Unemotional Traits (ICU; Essau et al., 2006)

The ICU contains 24 items with three factors: callousness (11 items), uncaring (eight items) and unemotional (five items). Each item is rated on a four-point Likert scale, ranging from 1 (“Not at all true”) to 4 (“Definitely true”). The higher score indicated a higher endorsement of the item characteristic. The Chinese version of the ICU was created and validated in a sample of Chinese community adults (Wang et al., 2017b), and in that study the Cronbach’s αs were 0.80, 0.75, 0.68, and 0.66 for the total score breakdown of callousness, uncaring, and unemotional, respectively.

#### Antisocial Process Screening Device – Self-Report Version (APSD-SR; Frick and Hare, 2001)

The APSD-SR is a 20-item scale that assesses antisocial behaviors and psychopathic traits in youth. It has three main factors: callous/unemotional (six items), narcissism (seven items) and impulsivity (five items). Each item is rated on a three-point Likert scale from 0 (“Not at all true”) to 2 (“Definitely true”). As prior studies with justice-involved youths validated (e.g., Murrie and Cornell, 2002; Pardini et al., 2003), Cronbach’s αs ranged from insufficient to acceptable in the current study, 0.71 for the total, 0.44 for the callous-unemotional dimension, 0.61 for the impulsivity dimension, and 0.55 for the narcissism dimension.

#### Youth Psychopathic Traits Inventory – Short Version (YPI-S; van Baardewijk et al., 2010)

The YPI-S is an 18-item self-report questionnaire that assesses the core psychopathic personality traits (Andershed et al., 2002; Wang et al., 2017a). It consists of three factors: interpersonal (grandiose-manipulative), affective (callous-unemotional), and behavioral (impulsive-irresponsible). Each factor has eight items and each item is scored on a four-point Likert scale ranging from 1 (“Does not apply at all”) to 4 (“Applies very well”). Cronbach’s αs in the present study were 0.79 for the YPI-S total, 0.76 for the interpersonal scale, and 0.70 for the behavioral scale, but somewhat low (i.e., 0.55) for the affective scale generally consistent with relevant findings (Colins et al., 2012).

#### Reactive-Proactive Aggression Questionnaire (RPQ; Raine et al., 2006)

The RPQ is a 23-item measure of proactive and reactive aggression in youth and young adults. Reactive aggression is assessed by 11 items, and proactive regression is assessed by 12 items. Each item is rated on a three-point scale from 0 (“Never”) to 2 (“Often”). In the present study, Cronbach’s αs for the total and factors were 0.94, 0.87, and 0.90, respectively.

#### Basic Empathy Scale (BES; Jolliffe and Farrington, 2006)

The BES is a 20-item scale that assesses empathy in juveniles. It has two factors: affective empathy (11 items) and cognitive empathy (nine items). Each item is scored on a five-point Likert scale ranging from 1 (“Strongly disagree”) to 5 (“Strongly agree”). In the present study, Cronbach’s αs for BES total and the two factors (affective and cognitive empathy scales) were 0.74, 0.68, and 0.76, respectively.

Based on standard translation procedures, all above-mentioned measures were adapted and translated into Mandarin Chinese, then back-translated into English by a team led by the second author who is skilled in both Mandarin Chinese and English. Differences in the original and the back-translated versions were discussed and solved by joint agreement of all translators to ensure accuracy.

### Data Analysis Strategy

Confirmatory factor analyses were carried out in Mplus 7.4 (Muthén and Muthén, 1998–2015). The factor models examined included the original ICU inter-correlated three-factor model (M1), the original ICU three-factor bifactor model (M2), the ICU-12 two-factor model (M3), and the ICU-11 two-factor model (M4). The robust weighted least-squares with a mean and variance adjustment (WLSMV) estimator was used to account for the categorical nature of the responses (Flora and Curran, 2004). To assess the model fit, we examined fit indices including chi-square (χ^2^), root mean square error of approximation (RMSEA), the Tucker-Lewis index (TLI), and the comparative fit index (CFI). A value of the TLI and CFI at 0.90 or higher and a value of RMSEA at 0.06 or smaller indicate a satisfactory model fit (Kline, 2010).

The internal consistency of the models were assessed by computing Cronbach’s α values as well as the mean inter-item correlations (MIC), a more straightforward indicator regardless of the length of a scale. Conventional guidelines suggest that the Cronbach’s α values ≥ 0.70 indicate acceptable internal consistency (Barker et al., 1994) and a MIC value between 0.15 and 0.50 indicates satisfactory internal consistency (Clark and Watson, 1995). To provide a more rigorous evaluation of the internal reliability of the ICU versions based on CFA models, we also investigated the composite reliability of the measurement properties of the scale. A value greater than 0.60 is generally considered acceptable (Bagozzi and Yi, 1988; Diamantopoulos and Siguaw, 2000). The convergent and discriminant validity evaluated via Pearson’s correlations were between the ICU scores and criterion variables (e.g., APSD-SR, YPI-S, RPQ and BES). We analyzed the internal consistency and correlations of the models using the SPSS program (IBM, SPSS version 19, 2010). Finally, the method proposed by Dunn and Clark (1969) was used (see Steiger, 1980 for more details)^1^ to determine whether the strength of the correlations with criterion measures differed between the original ICU and the best-fit model of ICU.

## Results

Table 1 reports descriptive statistics including means, standard deviations, number of items as well as Cronbach’s α values and MICs about all variables in the currents study.

**TABLE 1:** Descriptive statistics and reliability estimates for all variables.

	**Mean**	**SD**	**MIC**	**α**	***N***
**ICU-24**					
Unemotional	13.36	2.48	0.13	0.41	5
Callousness	18.64	4.99	0.25	0.77	11
Uncaring	17.78	4.70	0.35	0.81	8
Total	49.78	8.28	0.13	0.77	24
**ICU-12**					
Callousness	11.26	3.59	0.29	0.73	7
Uncaring	10.95	3.19	0.35	0.73	5
Total	22.20	5.20	0.19	0.73	12
**ICU-11**					
Callousness	9.02	3.19	0.34	0.75	6
Uncaring	10.95	3.19	0.35	0.73	5
Total	19.96	5.02	0.22	0.75	11
**APSD-SR**					
Impulsivity	3.35	2.24	0.24	0.61	5
CU	3.69	2.02	0.13	0.44	6
Narcissism	3.47	2.28	0.16	0.55	7
Total	11.71	5.26	0.11	0.71	20
**YPI-S**					
Behavioral factor	11.31	3.69	0.34	0.76	6
Affective factor	10.70	3.04	0.17	0.55	6
Interpersonal factor	8.80	2.75	0.30	0.70	6
Total	30.71	7.14	0.18	0.79	18
**RPQ**					
Reactive	6.84	4.63	0.39	0.87	11
Proactive	5.03	5.00	0.43	0.90	12
Total	11.84	9.13	0.39	0.94	23
**BES**					
Affective	34.69	6.04	0.16	0.69	11
Cognitive	33.50	5.32	0.26	0.76	9
Total	68.26	8.79	0.12	0.74	20

*ICU-24, Inventory of Callous and Unemotional Traits; ICU-12, Inventory of Callous and Unemotional Traits – 12 items, short version; ICU-11, Inventory of Callous and Unemotional Traits – 11 items, short version; APSD-SR, Antisocial Process Screening Device – self-report version; CU, Callous-Unemotional Traits; YPI-S, Youth Psychopathic Traits Inventory – short version; RPQ, Reactive-Proactive Aggression Questionnaire; BES, Basic Empathy Scale; SD, standard deviation; MIC, mean inter-item correlation; *N*, number of items.*

### Confirmatory Factor Analysis

Table 2 shows the fit indices of competitive models used in the current study. Fit indices showed an unacceptable fit for the inter-correlated three-factor model (M1; χ^2^ = 1901.46, df = 249, CFI = 0.71, TLI = 0.68, RMSEA = 0.10) and for the original three-factor bifactor (M2; χ^2^ = 1930.16, df = 228, CFI = 0.70, TLI = 0.64, RMSEA = 0.11). The two-factor model of the ICU-12 had significantly better fit than the M1 or M2, but the fit indices were still unsatisfactory (CFI < 0.90, TLI < 0.90, RMSEA > 0.80). Moreover, Item Six had the lowest loading (λ = 0.26, see Table 3). The two-factor model (ICU-11) that excluded Item Six had an excellent fit (χ^2^ = 149.77, df = 43; CFI = 0.95, TLI = 0.94, RMSEA = 0.06).

**TABLE 2:** Goodness-of-fit indices for the different models of ICU.

	**WLSMVχ^2^**	**df**	**RMSEA (90% CI)**	**CFI**	**TLI**
M1	1901.46^∗∗∗^	249	0.10 [0.10, 0.11]	0.71	0.68
M2	1930.16^∗∗∗^	228	0.11 [0.11, 0.12]	0.70	0.64
M3	302.34^∗∗∗^	53	0.09 [0.08, 0.10]	0.89	0.87
M4	149.77^∗∗∗^	43	0.06 [0.05, 0.08]	0.95	0.94

*M1, inter-correlated three-factor model; M2, original three-factor bifactor model; M3, ICU-12; M4, ICU-11; WLSMV, weighted least squares with mean and variance; df, degrees of freedom; RMSEA, root mean square error of approximation; 90% CI, 90% confidence interval for RMSEA; CFI, Comparative Fit Index; TLI, Tucker–Lewis Index. ^∗∗∗^*p* < 0.001.*

**TABLE 3:** Factor loadings for the relatively good fit two-factor model for ICU-12 and ICU-11.

**Items**	**Callousness**	**Uncaring**
(4) I do not care who I hurt to get what I want	0.72/0.72	
(6) I do not show my emotions to others	0.26	
(9) I do not care if I get into trouble	0.75/0.74	
(11) I do not care about doing things well	0.60/0.59	
(12) I seem very cold and uncaring to others	0.65/0.62	
(18) I do not feel remorseful when I do something wrong	0.61/0.61	
(21) The feelings of others are unimportant to me	0.78/0.79	
(5) I feel bad or guilty when I do something wrong (R)		0.73/0.74
(8) I am concerned about the feelings of others (R)		0.64/0.64
(16) I apologize (say “I am sorry”) to persons I hurt (R)		0.74/0.73
(17) I try not to hurt others’ feelings (R)		0.58/0.58
(24) I do things to make others feel good (R)		0.56/0.56

*ICU-12, Inventory of Callous-Unemotional Traits – 12 items, short version; ICU-11, Inventory of Callous-Unemotional Traits – 11 items, short version; (R), negatively worded items reverse-scored prior to analysis; factor loadings of ICU-11 are presented after the slash; all factor loadings are significant at a level of 0.001.*

With regards to the internal consistency, the Cronbach’s αs (MICs) for the ICU-11 total score, the callousness factor and uncaring factor were 0.75 (MIC = 0.22), 0.75 (MIC = 0.34), and 0.73 (MIC = 0.35), respectively. Furthermore, the results showed that all factor scores of the ICU-11 were measured with satisfactory composite reliability (total score, ρ_*c*_ = 0.90; callousness, ρ_*c*_ = 0.84; uncaring, ρ_*c*_ = 0.79). The correlation between the two factors was.24 (*p* < 0.001) at the observed level and 0.21 (*p* < 0.001) at the latent variable level, indicating a relatively weak intercorrelation.

### Convergent and Criterion Validity

Table 4 shows Pearson’s correlations between the ICU-11 and external criterion measures. As expected, there were significantly positive correlations between the ICU-11 factors and APSD-SR factors. The ICU-11 uncaring factor had a strong correlation with the APSD-SR callous/unemotional factor (*r* = 0.50, *p* < 0.001). The ICU-11 callousness factor was strongly correlated with the APSD-SR impulsiveness factor as well as the APSD-SR total (*r* = 0.50 and 0.53, *p*s < 0.001, respectively). The ICU-11 callousness factor showed significantly positive correlations with the YPI-S total scores and factors (*r*s = 0.45–0.67, *p*s < 0.001). On the other hand, the ICU-11 uncaring factor had weak correlations with the YPI-S behavioral factor and YPI-S total scores (*r* = 0.22, *p* < 0.001, and 0.11, *p* < 0.05, respectively), and was not significantly correlated with the YPI-S affective (*r* = −0.02, *p* > 0.05) or interpersonal factors (*r* = −0.04, *p* > 0.05).

**TABLE 4:** Pearson correlations of ICU-11, ICU-24, and their factors with relevant external variables.

		**ICU-11**			**ICU-24**			***Z***
	
**APSD-SR**	**Uncaring**	**Callousness**	**Total**	**Unemotional**	**Uncaring**	**Callousness**	**Total**	**ICU-24 Total vs. ICU-11 Total**
Impulsivity	0.16^∗∗∗^	0.50^∗∗∗^	0.42^∗∗∗^	–0.07	0.18^∗∗∗^	0.55^∗∗∗^	0.42^∗∗∗^	0.00
CU	0.50^∗∗∗^	0.29^∗∗∗^	0.50^∗∗∗^	–0.01	0.51^∗∗∗^	0.37^∗∗∗^	0.52^∗∗∗^	1.37
Narcissism	0.15^∗∗∗^	0.38^∗∗∗^	0.33^∗∗∗^	–0.12^∗∗^	0.13^∗∗^	0.42^∗∗∗^	0.29^∗∗∗^	−2.46^*^
Total	0.35^∗∗∗^	0.53^∗∗∗^	0.56^∗∗∗^	−0.10^*^	0.36^∗∗∗^	0.61^∗∗∗^	0.55^∗∗∗^	–0.71
**YPI-S scores**								
Behavioral factor	0.22^∗∗∗^	0.58^∗∗∗^	0.50^∗∗∗^	–0.19^∗∗∗^	0.23^∗∗∗^	0.64^∗∗∗^	0.46^∗∗∗^	–2.68^∗∗^
Affective factor	–0.02	0.46^∗∗∗^	0.29^∗∗∗^	–0.04	–0.06	0.42^∗∗∗^	0.24^∗∗∗^	–3.03^∗∗^
Interpersonal factor	–0.04	0.45^∗∗∗^	0.26^∗∗∗^	–0.08	–0.08	0.40^∗∗∗^	0.17^∗∗∗^	–5.39^∗∗∗^
Total	0.11^*^	0.67^∗∗∗^	0.49^∗∗∗^	–0.14^∗∗^	0.08	0.66^∗∗∗^	0.41^∗∗∗^	–5.28^∗∗∗^
**RPQ scores**								
Reactive	0.21^∗∗∗^	0.47^∗∗∗^	0.43^∗∗∗^	–0.21^∗∗∗^	0.21^∗∗∗^	0.52^∗∗∗^	0.37^∗∗∗^	–3.84^∗∗∗^
Proactive	0.21^∗∗∗^	0.50^∗∗∗^	0.44^∗∗∗^	–0.14^∗∗^	0.20^∗∗∗^	0.52^∗∗∗^	0.39^∗∗∗^	–3.22^∗∗^
Total	0.23^∗∗∗^	0.51^∗∗∗^	0.46^∗∗∗^	–0.18^∗∗∗^	0.22^∗∗∗^	0.55^∗∗∗^	0.41^∗∗∗^	–3.26^∗∗^
**BES scores**								
Affective	–0.32^∗∗∗^	–0.24^∗∗∗^	–0.35^∗∗∗^	−0.09^*^	–0.25^∗∗∗^	–0.24^∗∗∗^	–0.32^∗∗∗^	1.86
Cognitive	–0.39^∗∗∗^	–0.30^∗∗∗^	–0.45^∗∗∗^	0.08	–0.38^∗∗∗^	–0.35^∗∗∗^	–0.42^∗∗∗^	1.95
Total	–0.45^∗∗∗^	–0.35^∗∗∗^	–0.51^∗∗∗^	–0.02	–0.40^∗∗∗^	–0.38^∗∗∗^	–0.47^∗∗∗^	2.70^∗∗^
Age	–0.06	–0.11^∗∗^	–0.12^∗∗^	–0.05	–0.08	–0.12^∗∗^	–0.14^∗∗^	–1.17
AIJDC	0.08	0.12^∗∗^	0.13^∗∗^	–0.01	0.10^*^	0.11^∗∗^	0.13^∗∗^	0.00
DI	–0.14^∗∗∗^	–0.21^∗∗∗^	–0.23^∗∗∗^	–0.05	–0.16^∗∗∗^	–0.22^∗∗∗^	–0.25^∗∗∗^	–1.20

ICU-24, Inventory of Callous and Unemotional Traits; ICU-11, Inventory of Callous and Unemotional Traits – 11 items, short version; APSD-SR, Antisocial Process Screening Device – self-report version; CU, Callous-Unemotional Traits; YPI-S, Youth Psychopathic Traits Inventory – short version; RPQ, Reactive-Proactive Aggression Questionnaire; BES, Basic Empathy Scale; AIJDC, Age of incarceration into a Juvenile Detention Center; DI, duration of incarceration. ^*^p < 0.05, ^∗∗^*p* < 0.01, ^∗∗∗^*p* < 0.001.

The ICU-11 total score and the ICU-11 callousness scale were moderately and positively correlated with two kinds of aggression assessed by RPQ (see Table 4). On the other hand, the ICU-11 uncaring scale showed weak associations with aggression (*r*s < 0.30). The ICU-11 total also had a significant negative correlation with empathy as measured by the BES (total BES: *r* = −0.51, *p* < 0.001; affective factor: *r* = −0.35, *p* < 0.001; cognitive factor: *r* = −0.45, *p* < 0.001). The ICU-11 uncaring factor had stronger relationships with the BES and its factors (*r*s = −0.32 to −0.45, *p*s < 0.001) than the ICU-11 callousness factor did (*r* = −0.24 to -0.35, *p*s < 0.001).

Correlations between the original ICU total and factor scores and external variables were similar to those for the ICU-11 (see Table 4). The unemotional factor of the original ICU demonstrated weaker or no associations at all with the external variables, whereas it showed robustly stronger associations with scores for reactive aggression, the YPI-S behavioral factor, proactive aggression and the APSD-SR narcissism factor.

Table 4 also presents the correlations between the ICU-11 and other variables (e.g., age, age of incarceration into a juvenile detention center). The ICU-11 and subscale scores were negatively correlated with age, but positively correlated with the age of incarceration. To explore this further, we inspected the correlations between the ICU-11 and the duration of incarceration (i.e., difference between current age and first arrest age). There was a significant negative correlation between the ICU-11 and the duration of incarceration, suggesting that participants with a longer stay at the center reported lower ICU scores. The original ICU were as and the ICU-11 had similar correlations with those variables.

Next, we compared the ICU-11 and the original ICU in terms of their correlations with the external criterion variables. *Z* values (*p* < 0.01, two-tailed for significance) were calculated based on Dunn and Clark (1969) method (see Table 4). For most variables, the ICU-11 total showed stronger correlations to the external criterion than the ICU-24 did.

## Discussion

The present study is the first study that investigated the factor structure and psychometric properties of the ICU in Chinese detained youth samples. Consistent with previous studies using samples of Chinese community adults (Wang et al., 2017b) and children (Wang et al., 2019), the three-factor model of the original ICU was not replicated in the present study, but the ICU-11 with a two-factor model was found to have the best fit for the data. The reliability coefficients of the ICU-11 and its factors were also more satisfying than those of the original ICU. Finally, the convergent validity of the ICU was demonstrated by significant correlations between the ICU-11 and a range of criteria variables.

Previous studies of the ICU using Western samples found that the three-factor bifactor model received the most support in adolescents (Kimonis et al., 2008; Pihet et al., 2015). However, the bifactor model could not be replicated in the current study as well as it could with other Chinese samples (Wang et al., 2017b). The poor fit was mainly attributed to the low factor loading of items on the unemotional factor. Additionally, the unemotional factor of the original ICU-24 showed substantially low Cronbach’s α value and poor validity, which was in line with previous studies (Kimonis et al., 2008; Byrd et al., 2013; Wang et al., 2017b; Deng et al., 2019). Despite the unemotional factor showing high association with empathy and modest association with proactive aggression across over ten studies (Cardinale and Marsh, 2017), these findings were hardly replicated in this Chinese detained juvenile sample thus to some extent indicated the unemotional were not a stable indicator of the construct of CU traits and needed further validation.

These results have reinforced the idea that the original unemotional factor of the ICU might not be a reliable construct in detained youth, at least when using the self- or other-report versions of the ICU. A major reason for this is considered to be that the affective deficits lack accurate descriptions, and that most items looking at the unemotional factor refer to the outward expression of emotions rather than the experience of them, both of which result in poor internal consistency in the unemotional factor (Cardinale and Marsh, 2017). The features of unemotional trait are mostly negative, which are more difficult to detect for both the subjects and the observers. Subjects may not be aware of the absence of emotion, while observers may mistake the symptoms as the subject being shy or introverted. Another factor is that the expressions of “unemotional” characteristics could also be contributed to by other constructs, such as social expectations or problematic emotional expressions (such as those by autistic children). Social expectations vary greatly across cultures and, thus, can negatively influence the multigroup measurement invariance across the original English samples, as well as subsequent samples from other cultural groups. All these issues could result in lower reliability of the unemotional factor.

With regards to problematic emotional expressions, previous studies have consistently found negative correlations of the unemotional factor with aggression assessments (Wang et al., 2017b). Subjects with abnormal emotional regulation and expression may externalize emotions such as anger, demonstrating aggressive behaviors. Taken together, the items of the unemotional factor may be tapping into a construct departing from CU. Further research into the unemotional factor is warranted.

The shortened ICU-12 that excluded most items from the unemotional factor achieved a better fit than the original ICU factor structures, with the exception of Item 6, which had a low factor loading. This was consistent with previous studies (Colins et al., 2016; Wang et al., 2017b, 2019). After removing Item 6, the ICU-11 had the best fit for the current data.

The analysis of the internal consistency of the ICU-11 revealed mostly good to extremely good values, with most values exceeding both the recommended minimum Cronbach’s α of 0.70 and the recommended minimum composite reliability of 0.60, as well as the MICs in a favorable range (>0.19). The Cronbach’s α values of both the ICU-12 and the ICU-11 uncaring factors in the present study were greater than in previous findings (Wang et al., 2017b, 2019). The greater factor reliability could be due to the fact that the sample for this study had an older average age than studies where the sample consisted of children. Adolescent subjects in the present study might have had better reading comprehension than those under the age of 12 years (Soto et al., 2008; Deng et al., 2019). In addition, the ICU was developed based on a clinical sample, thus could be more precise when measuring CU traits among subjects who were on the high end of the latent traits. And, in comparison to community samples, the detention environment helped to guarantee the standardization of the testing process, which may have offered more consistent responses to the ICU items. Furthermore, it was worth mentioning that the α values for ICU scores in clinical samples had been proven to be more variable than in non-clinical samples (Deng et al., 2019). More evidence for internal consistency of ICU-11 in Chinese clinical samples is needed in the future.

With regards to external validity, the ICU-11 demonstrated the expected correlations with the criterion variables (i.e., APSD-SR, YPI-S, and RPQ), and the pattern of correlations were similar to those of the original ICU.

As reported by previous findings of a meta-analytic review (Cardinale and Marsh, 2017), strong associations were found between psychopathy and the total ICU-11, callousness factor and uncaring factor, and the callousness factor compared with the uncaring factor displayed stronger associations with measures of psychopathy in detained samples. Specifically, the directions and magnitudes of the correlations between the ICU and the YPI-S were comparable with those reported in previous studies (Roose et al., 2010; Pihet et al., 2015). Most correlations found between the ICU-11 scales and APSD-SR scales were higher than those reported in Wang et al. (2017b), which reflects the different demographics of the two samples. Wang et al. (2017b) used a community sample, in which the manifest of antisocial personality had a limited range.

Meanwhile, consistent with previous studies, the aggression factor showed a stronger correlation with callousness than with the uncaring factor. Kimonis et al. (2008) suggested that this could be due to the fact that callousness has a greater comorbidity with aggression, whereas uncaring was expressed through their offences committed. The ICU-11 also demonstrated expected negative associations with empathy when assessed by the BES (e.g., Kimonis et al., 2008; Roose et al., 2010). Dolan and Fullam (2006) suggested that the temperamental fearlessness featured in CU traits can result in a decrease in the arousal of the autonomic nervous system. This in turns leads to difficulties in recognizing others’ emotional distress among individuals who rank high in psychopathy measurements. The uncaring factor also had stronger correlations with the BES than the callousness, suggesting that the uncaring is a major component in one’s inability to recognize others’ emotions. Similar findings were also reported by Pechorro et al. (2016a, 2017).

We also evaluated how the CU traits were related to subjects’ age, age of incarceration, and the duration of incarceration. Inconsistent with previous findings (Byrd et al., 2013; Pechorro et al., 2017), we found that the CU traits had moderately negative associations with participants’ age and the duration of incarceration. This suggested that older participants might be better at identifying and reporting emotion. In addition, Asscher et al. (2011) indicated that individual age when assessing psychopathy played a moderating role in the associations between psychopathy and delinquency. Notably, during the course of childhood to adolescence, individuals with psychopathic traits likely have learned to conceal their cognitive empathy deficits or the relevant empathy skills may have improved (Dadds et al., 2009). Thus, the strength of association between psychopathy and delinquency diminished with increasing age (Asscher et al., 2011). Overall, the incarceration confinement and education seemed to have a positive effect on transforming the pathological personality of the juvenile offenders.

Summarizing, prior findings have emphasized the importance of CU traits which appear to mirror several related aspects about affective and interpersonal functioning (Lynam et al., 2005). CU traits also provide evidence to designate and understand severely antisocial youths, especially the adolescent offenders who had great risk in subsequent violent offenses throughout a 2-year period after releasing from incarceration (Vincent et al., 2003). Currently in China, market reforms have promoted the social transition, meanwhile, the crime rate of juveniles has assumed the trend of escalation and criminal nature of the case has become more and more serious. Assessment of CU traits with the ICU particularly the shortened ICU-11 thus remains a significant research focus with crucial clinical implications in Chinese juvenile offenders. Specifically, extant findings may allow psychological staff to tap Chinese detained boys the existence of the common factor, analyze the causes of crime or delinquency and thus take appropriate measures to improve the system of current criminal penalty.

### Limitations

Several limitations must be acknowledged. First, the current sample was made up only of males, making it unclear how the results can be generalized toward female detention populations. Pechorro et al. (2017) found manifestations of generalized problem conducts in female juveniles with CU traits might depend on the criminal justice system. Future study should look at female populations and examine potential gender differences regarding the validity and reliability of the ICU. Second, all measures were based on self-reporting and the current study did not explore the detailed offending history of the detained boys, which easily demonstrated method variance and might inflate relations among study variables. Future research should consider the inclusion of multiple methods of data gathering, such as interviews, multiple-informant formats, such as caregiver- or caseworker-reported, and include more delinquent details from case records. Third, the current study had a cross-sectional design, which restricted the conclusions on the predictive utility of ICU traits, as well as any causal inferences. Future longitudinal studies should be conducted that evaluate correlations over time. Finally, future research also should investigate the relationships between the ICU-11 and variables such as delinquent histories, conduct disorder, age of first contact with the law, and the severity of the crime.

## Conclusion

The current study is the first study to explore the factor structure and construct validity of the ICU in a large Chinese male juvenile offender sample. Consistent with previous studies looking at Chinese samples (Wang et al., 2017b, 2019), CFA analyses indicated that the ICU-11 with two factors had the best model fit. Both the total and two factors’ scores showed acceptable internal consistency. The results also demonstrated promising convergent validity of the ICU-11. Overall, the current study’s findings suggest that the ICU-11 holds promise as an informative alternative for the original ICU form, particularly in detained Chinese male youths.

## Data Availability

The datasets generated for this study are available on request to the corresponding author.

## Ethics Statement

After receiving written informed consent from the detainees’ parents or caregivers, the detainees were informed about the aims, content, and duration of the study by trained research assistants. The study duration was approximately 40 min. This study was approved by the Human Subjects Review Committee at Guangzhou.

## Author Contributions

XZ, YS, CZ, JL, and WY made substantial contribution to the analysis and interpretation of the data, drafted the manuscript, provided the final approval for the manuscript, and agreed to be accountable for all aspects of the work in ensuring that questions related to the accuracy or integrity of any part of the work are appropriately investigated and resolved. M-CW and YG made substantial contributions to the conception and the design of the study, drafted the manuscript, provided final approval for the manuscript, and agreed to be accountable for all aspects of the work in ensuring that questions related to the accuracy or integrity of any part of the work are appropriately investigated and resolved.

## Conflict of Interest Statement

The authors declare that the research was conducted in the absence of any commercial or financial relationships that could be construed as a potential conflict of interest.

## Footnotes

1 Using a spreadsheet that was developed by DeCoster and Iselin (2005) and can be retrieved at: http://stat-help.com/spreadsheets.html
